# Diseases of the Urinary Apparatus; Phlegmasic Affections

**Published:** 1892-12

**Authors:** 


DISEASES OF THE URINARY APPARATUS; PHLEGMASIC AFFECTIONS. By JOHN W. S. GOULEY, M. D., Surgeon to Bellevue Hospital. New York: D. Appleton & Co. 1892.

Dr. Gouley has revised, and D. Appleton & Co. have published, his lectures relating to diseases of the urinary apparatus, delivered before the class of the Bellevue Hospital Medical College during the year 1891.

A brief *résumé* of the early history of the practice of this branch of the surgical art is given in the introduction of this work, in which it is related that prosthectomy was commonly practised by several ancient nations, for the prevention of disease, two thousand years ago. Gouley might have recorded that prosthectomy is crudely self-performed by the youth of a savage African tribe for the purpose of gaining favor with the females, and with no thought of sanitary benefit. Again, it is related that another savage tribe execute external urethrotomy in the perineal region for the purpose of making a new exit of escape for the seminal fluid, and thus avoiding fructification. This plan is less harmful than some of the ways and methods practised by civilized races, who profane the sacred injunction to increase, multiply, and replenish the earth.

The urethral tract is divided into six parts, as follows: Prostatic, membranous, perineal, scrotal, phallic, and balanic.

The general pathology is divided sixteen times, as follows: Phlegmasic, stenotic, auxetic, echmatic, ectatic, lithic, neoplastic, adenic, blastomatic, cystic, entozoic, toxic, traumatic, allotrylic, teratic, and functional affections.

The following-named larvæ are alleged invaders of the urinary tract, namely: spiroptera hominis, diplosoma crenatum, dactylus aculeatus, as also are the following-named parasites: echinococcus homnis, distoma hematobium, pentastoma denticulatum, strongylus gigas, filaria sanguinis, trichina spiralis, and Gouley might have added pin-worms.

Diseases of the Urinary Apparatus is severely technical and classic, and reminds the reviewer of his attempt to read Rokitansky's Pathology, as a side line, when a first course student.

Dr. Gouley avers: "Nothing, so far discovered, has sufficed to explain the nature of the contagium of that variety of urethritis, miscalled gonorrhea." Again, under the heading Methodical Treatment, he says: "To treat urethritis methodically and rationally, it is necessary to ascertain the nature, cause, etc., of the phlegmasic attack." How are we to treat the malady "methodically and rationally" when such able authority as Dr. Gouley affirms that its nature and cause have not been sufficiently explained?

Abortive treatment is justly condemned. The administration of balsams, local treatment by injections or irrigation during the second or acute inflammatory stage, is approved. Unfortunately, in spite of the numerous methods of treatment, in defiance of the numberless remedies and formulæ proposed in text-books and periodicals for the relief of this disease, numerous urethræ continue to weep. More, and worse even than this, is the infection of innocent persons by the contagion, borne and transmitted by cases of unrelieved specific urethritis. Why not hold to the good old name, "clap," made classic by long use? If the teaching of Noeggerath, Price, Sinclair, (BUFFALO MEDICAL JOURNAL, page 488, March, 1891,) and many other eminent authorities is accepted, this is the most persistent, insidious, and virulent malady which afflicts mankind, and the body politic would be warranted in prescribing quarantine and a yellow flag for all cases of specific urethritis, whether affecting the urethra, vagina, uterus, or Fallopian tubes.

The theory of Neisser, that microbes (gonococci) are the cause of specific phlegmasiæ of the genito-urinary mucosa, was favorably received by the profession, and we had pinned our faith to it. By turning our microscope on the secretions from the morbid tracts, and finding gonococci, our diagnosis was certain; Dr. Gouley appears upon the scene, shatters our faith, and we are at sea again. Still, we must confess that the discovery of gonococci and the claim that these microbes are the cause of gonorrhea, or specific urethritis, has not led to additional successful treatment of this malady. Gorging the urethra with microbicides has not produced any more satisfactory results than the empirical methods which formerly prevailed, for the morning tear yet greets the waking hour.

The germs escape their enemy by dodging behind the epithelium and burying themselves in the submucous structures, and also find safety in the mucous crypts and glandular openings of the genital tract, where they grow fat on the products of phlegmasiæ produced by irritant, germicidal remedies. We fondly hoped that at last we had found a method of exact diagnosis, and that by following its wake we would have rational and exact methods of treatment.

Diseases of the Urinary Apparatus is an able and scholarly production, and we only regret that differential diagnosis cannot be more positively made, and treatment more effectual and certain. It is evident that we have not yet reached the dawn of a venereal millenium. B. H. D.

